# Inadvertent Subdural Injection during Cervical Transforaminal Epidural Steroid Injection

**DOI:** 10.1155/2013/847085

**Published:** 2013-12-31

**Authors:** Kesavan Sadacharam, Jeffrey D. Petersohn, Michael S. Green

**Affiliations:** ^1^Drexel University College of Medicine, Hahnemann University Hospital, Philadelphia, PA 19102, USA; ^2^Drexel University College of Medicine, Pain Care PC, Linwood, NJ 08221, USA; ^3^Department of Anesthesiology, New College Building, Room 7502, 245 North 15th Street, Mail Stop 310, Philadelphia, PA 19102, USA

## Abstract

Serious complications following cervical epidural steroid injection are rare. Subdural injection of local anesthetic and steroid represents a rare but potentially life threatening complication. A patient presented with left sided cervical pain radiating into the left upper extremity with motor deficit. MRI showed absent lordosis with a broad left paramedian disc-osteophyte complex impinging the spinal cord at C5-6. During C5-6 transforaminal epidural steroid injection contrast in AP fluoroscopic view demonstrated a subdural contrast pattern. The needle was withdrawn slightly and repositioned. Normal lateral epidural and nerve root contrast pattern was subsequently obtained and injection followed with immediate improvement in radicular symptoms. There were no postoperative complications on subsequent clinic follow-up. The subdural space is a potential space between the arachnoid and dura mater. As the subdural space is larger in the cervical region, there may be an elevated potential for inadvertent subdural injection. Needle placement in the cervical subdural space during transforaminal injection is uncommon. Failure to identify aberrant needle entry within the cervical subdural space may result in life threatening complications. We recommend initial injection of a limited volume of contrast agent to detect inadvertent subdural space placement.

## 1. Introduction

Cervical radiculopathy is a common condition affecting 83 per 100,000 persons each year [1]. The cause varies by patient age with intervertebral disc herniation common in younger persons and spondylosis predominating in the older age group. Cervical spondylosis is present in 75% of patients older than 65 years of age. Both pathologies can produce anatomic stenosis resulting in impingement or compression of nerve roots or spinal cord. Presenting symptoms include cervical pain with or without radiculopathic upper extremity pain, weakness, deep tendon reflex depression, headache, or vertigo. Duration and severity of symptoms directs selection of treatment modality. Common conservative treatment consists of nonsteroidal anti-inflammatory drugs, anticonvulsants, muscle relaxants, and physical therapy. Efficacy of cervical epidural steroid injection for patients unresponsive to conservative treatment has been demonstrated [2–4]. Symptoms refractory to interventional care or demonstrating myelopathic symptoms of spinal cord compression necessitate consideration for surgical intervention.

Epidural injection in the cervical spine can be performed by interlaminar or transforaminal approaches. Transforaminal injection provides the most direct route for delivery of medication to the lateral recess or foraminal pathology. As low resistance to liquid flow is expected in the dorsal epidural space compared with high flow resistance imposed by lateral recess or foraminal narrowing, interlaminar approaches may fail to deliver adequate medications to pathology located distally in the anterolateral epidural space or within the radicular canal. Potentially superior outcomes support use of the transforaminal approach [4–7].

Derby et al. report the overall rate of complication following cervical epidural steroid injections as 5 per 1000 injections [8]. Complications described as minor include vasovagal episodes, rashes, headache, worsening pain, new paresthesia, and pain. Rare major complications include direct spinal cord trauma, epidural hematoma or abscess, transection of vertebral artery, and injection of particulate steroid into a radiculomedullary artery or vertebral artery resulting in spinal cord or posterior cerebellar embolic infarction [9, 10]. Subdural injection of local anesthetic and steroid represents a rare but potentially life threatening complication. The incidence of subdural injection is 0.8% with lumbar epidural injection [11] and surprisingly high 1.6–3.2% during diagnostic myelography [12]. We present a case where expeditious recognition of subdural space injection allowed adjustment of technique and avoided a potentially deleterious patient outcome.

## 2. Case Description

A 37-year-old, right-handed female presented with left sided cervical pain radiating into the left upper extremity with ipsilateral fourth and fifth digit numbness and complaint of left upper extremity weakness. Her pain followed a motor vehicle accident one year prior to interventional pain consultation. Extensive unhelpful conservative treatments including physical therapy, chiropractic care, and pharmacologic intervention preceded her visit. Examination showed painless cervical flexion and extension to 50 and 40 degrees, respectively. Left sided cervical pain occurred with lateral cervical rotation to both the left and right. Anterior foraminal tenderness was noted at left C6 and C7 nerve levels with positive Tinel's phenomenon radiating pain into the extremity. Hypoesthesia was noted in left C6 and C7 dermatomes with decreased motor power on left elbow extension, flexion, and forearm pronation. Deep tendon reflexes were intact and symmetrical. Radiographs demonstrated anterior osteophytes at C5-C6-C7. Cervical spine MRI showed absent lordosis with a broad left paramedian disc-osteophyte complex impinging the spinal cord at C5-6 and mild right C5-C6 neuroforaminal stenosis. There were chronic degenerative disc changes at C5-C6 and C6-C7. A left C5-6 transforaminal epidural steroid injection was planned in a monitored operating room setting.

**Figure 1:**
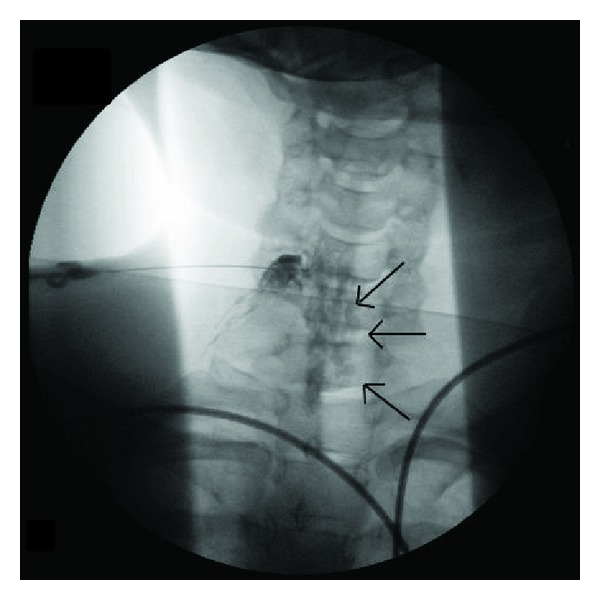
Anterior Posterior radiograph of the cervical spine showing subdural spread of dense contrast media. Multiple black arrows illustrate unusual contrast spread towards the midline.

**Figure 2:**
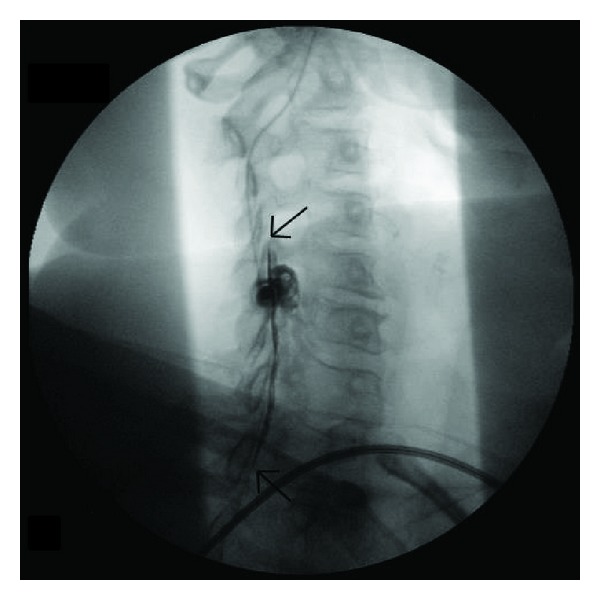
Oblique view of cervical spine with homogenous, high density, and elongated contrast outline limited to the dorsal aspect of the spinal canal indicating subdural spread. Arrow show the maximal extent of the unusual extensive cephalocaudal spread of contrast media with a sharp posterior border and a slightly wavy anterior border.

**Figure 3:**
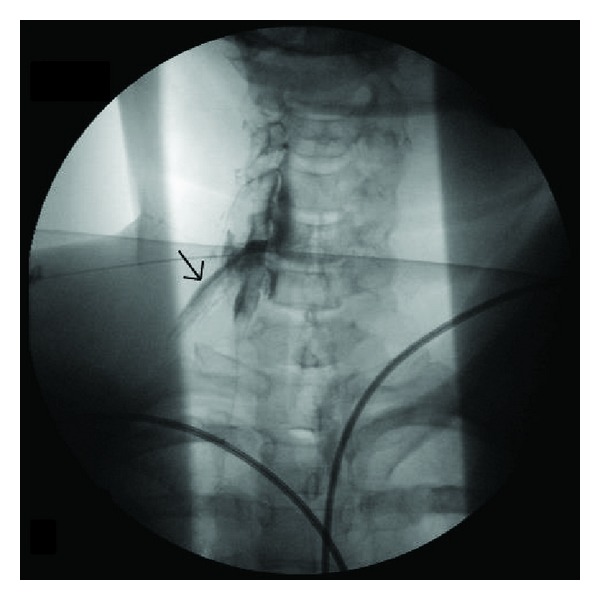
Repeat contrast injection following needle repositioning shows epidural injection with clear outline of nerve root, dorsal root ganglion, and lateral epidural space as demonstrated by arrow. Additional contrast is retained in the vertebral canal.

**Figure 4:**
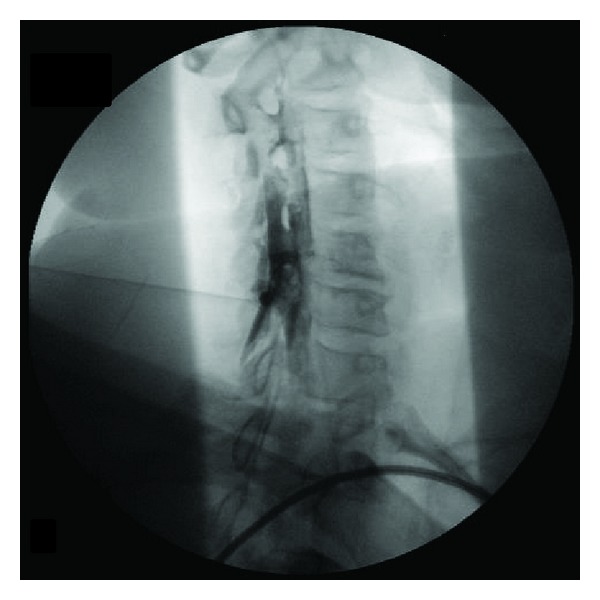
Oblique view after needle repositioning and repeat contrast injection shows the typical contrast pattern for epidural injection with limited cephalocaudal spread and outlining of nerve roots superimposed upon the extensive prior subdural contrast spread (seen here as caudally as T2).

The patient was placed in “park bench” position and following propofol based monitored anesthesia care, the left C5-6 neural foramen was visualized with anterior oblique fluoroscopic view and a 25 gauge 2.5 inch short bevel needle was advanced to enter the midneural foramen at the posterior border. Anterior posterior (AP) fluoroscopic views facilitated advancement of the needle tip to beneath the midpoint of the pedicle. Following negative aspiration of blood or CSF, injection of iohexol radiocontrast 0.3 mL demonstrated a broad homogenous density extending over about 1/3 of the ipsilateral vertebral canal in AP view (Figure 1). Oblique fluoroscopic view unexpectedly demonstrated extensive linear opacification along the posterior border of the epidural space from C5 to T1 (Figure 2). Inadvertent subdural injection was diagnosed. The needle was withdrawn slightly and repositioned with subsequent contrast injection demonstrating a satisfactory neurogram of the proximal nerve root and dorsal root ganglion with contrast entry in the lateral epidural space (Figures 3 and 4). Injection of bupivacaine 0.25% 0.5 mL and betamethasone 3 mg followed. Her radicular symptoms were improved when evaluated 15 minutes thereafter in the postanesthesia care unit. No immediate postoperative complaints of pain or headache existed. Office follow-up demonstrated an entirely uncomplicated course with complete resolution of cervical pain and radicular symptoms.

## 3. Discussion

The spinal cord and the spinal nerve proximal to the dorsal root ganglion are surrounded by a trilaminar structure composed of an outer layer of dense fibrous dura mater, a middle arachnoid layer of thin nonvascular tissue, and inner pial layer of thick vascular connective tissue. The dura-arachnoid interface is formed by the outer layer of the arachnoid membrane and inner layer of dura mater. The outer surface of the arachnoid mater consists of closely arranged cells with numerous tight junctions with minimal extracellular space. The compact and tightly arranged nature of the cells provides the effective barrier between cerebrospinal fluid and blood circulation in dura mater. In contrast, the inner layer of dura mater is characterized by cells with few cellular junctions, no extracellular collagen, and multiple enlarged extracellular spaces. Electron microscopy shows the dura-arachnoid interface to be filled with neuroepithelial cells interconnected by few intercellular junctions. The presence of neuroepithelial cells within the dura-arachnoid interface demonstrates that this is not a true potential space like the intrapleural space [12]. Vandenabeele et al. [11] used electron microscopy to show a natural close connection between the outer layer of arachnoid membrane and inner layer of dura mater, but the combination of loosely arranged cells of the inner surface of the dura mater, absence of collagen, and few intercellular junctions among neuroepithelial cells provides the potential for a cleavage plane with subsequent enlargement of the subdural space due to pressure or trauma from air or fluid injection [11, 12]. While the dura mater and arachnoid can be separated by gently rolling the tissue between the fingers, it is unknown whether separation of these layers, and hence enlargement of the subdural space, occurs more frequently in patients with a history of trauma.

As the subdural space is larger in the cervical region as compared to the lumbar region, the risk for inadvertent subdural injections may also be greater in the cervical region. The subdural space typically extends from the inferior border of the second sacral vertebra into the intracranial space unlike the epidural space which typically terminates at the foramen magnum. As the subdural space is widest in its lateral and dorsal aspects, liquid injected into the subdural space will fill the dorsal and lateral compartments first. Since the dorsal spinal cord structures are predominantly composed of sensory fibers as opposed to the anterior cord structures which contain motor and sympathetic tracts, the clinical presentation of subdural injection affecting the spinal cord tends to produce predominant symptoms of sensory neural blockade rather than symptoms of either motor or sympathetic block [13, 14].

The greatest hazard of subdural injection may be posed by injection of local anesthetic into the subdural space as the small volume of the subdural space may allow material to be injected transforaminally to reach intracranial structures leading to significant neurological and hemodynamic complications including loss of consciousness, severe hypotension, bradycardia, and cardiac arrest [15, 16]. While initial injection of local anesthetics into the subdural space may produce symptoms and signs similar to intrathecal injection, subtle differences are noted. Collier described four clinical criteria as diagnostic of subdural injection including moderate hypotension, slow onset of symptoms, progressive respiratory difficulty, and complete recovery in 2 hours' time [17]. Hypotension and apnea are moderate as sympathetic and motor fibers in the cervical spinal cord are less affected than the posterior sensory structures.

Cervical interlaminar epidural injections and transforaminal approaches to the epidural space must be performed with fluoroscopic guidance, in the author's opinion. Imaging in at least two planes, typically AP and either an oblique or lateral plane, is required to confirm initial needle position. Confirmation of adequate needle placement by injection of contrast media requires observation of the pattern of contrast flow under continuous fluoroscopy or by digital subtraction angiography. With correct placement of needle at the neural foramen, the nerve root and dorsal root ganglion should be outlined by contrast in the AP fluoroscopic view. In the lateral view, there may be anterior as well as posterior dispersion of contrast into the epidural space. An oblique image may show a ring of contrast surrounding the exiting nerve root. The potential for diagnostic ultrasound to demonstrate subdural injection has been reported in dogs and horses [18].

In the case of inadvertent subdural space injection, the AP fluoroscopic view will show a dense collection of contrast extending in a homogenous fashion towards the center of the vertebral canal but typically without outline of the lateral epidural space, exiting nerve root or dorsal root ganglion. The lateral fluoroscopic view will demonstrate a homogenous, high density, elongated contrast outline typically limited to the dorsal aspect of the spinal canal. Due to the small volume of the subdural space relative to the epidural space, the cephalocaudal contrast spread will extend over multiple vertebral segments, as opposed to the typical single level contrast dispersion seen with injection of the same volume into the epidural space. Practitioners should become aware of the expectable limited cephalocaudal extent of normal contrast flow when using small volumes (0.3–0.5 mL) of injected contrast. While the posterior outline of the injected subdural contrast may be linear due to the dura mater, the anterior border, if opacified by contrast, may appear irregular due to the arachnoid mater [19, 20]. Given identical volumes of contrast injection, the density of the subdural contrast will be far greater due to dilution of intrathecal contrast by CSF [19]. While the pattern of subarachnoid contrast changes with alteration in body position to reflect gravity, such migration is theoretically less likely with subdural injection.

Needle placement in the cervical subdural space during transforaminal injection is an uncommon event. Failure to identify aberrant needle placement by observation of the extent and nature of the radiocontrast dispersion may result in life threatening complications upon subsequent injection of steroid and local anesthetic into the subdural space. Interventional pain physicians must have an adequate knowledge of normal and abnormal patterns of epidural and perineural contrast dispersion and flow in order to avoid harm to patients. This complication probably occurs more often than is recognized and may lead not only to a lack of efficacy, but also to a potential hazard [21]. Thus, we recommend that physicians performing transforaminal cervical epidural injection routinely initially inject a limited contrast volume (0.3 mL) under continuous fluoroscopy with diligent assessment of biplanar contrast spread as a viable strategy to detect subdural injection.
